# Evaluation of the Functional Performance in Turned Workpieces: Methodology and Application to UNS A92024-T3

**DOI:** 10.3390/ma11081264

**Published:** 2018-07-24

**Authors:** Álvaro Gómez-Parra, Alfredo Sanz, Antonio J. Gámez

**Affiliations:** 1 Department of Mechanical Engineering and Industrial Design, Faculty of Engineering, University of Cadiz, Avenida de la Universidad de Cadiz, 10, Puerto Real, E-11519 Cadiz, Spain; antoniojuan.gamez@uca.es; 2 Department of Aerospace Materials and Manufacturing, Polytechnic University of Madrid, Plaza del Cardenal Cisneros, 3, E-28040 Madrid, Spain; asl@aero.upm.es

**Keywords:** turning, UNS A92024-T3, corrosion, surface integrity, Ra, residual stress, functional performance, ultimate tensile strength

## Abstract

Turning of light alloys as aluminum-based UNS A92024-T3 is broadly implemented in the manufacture of critical aircraft parts, so ensuring a good functional performance of these pieces is essential. Moreover, operational conditions of these pieces include saline environments where corrosion processes are present. In this paper, a methodology for the evaluation of the functional performance in turned pieces is proposed. Specimens affected and not affected by corrosion are compared. In addition, performance in service through tensile stress tests of these parts is considered. The results show that turning improves the functional performance of UNS A92024-T3 alloy and that corrosion can enhance the mechanical properties of this alloy.

## 1. Introduction

The evaluation of the performance of a manufacturing process is a complex task that can be better approached when four fundamental and complementary points of view are recognized: economical, energetic, environmental, and functional. In this context, the global process performance has been defined as the center of gravity of a tetrahedron defined by setting these four elements in its apexes [1].

In particular, the aeronautical industry considers high-performance manufacturing, even at the cost of a loss in economic performance, provided the process is enhanced from the energetic, environmental and especially, functional points of view [2,3,4]. Functionality can be understood as the state of health of the workpiece [5]. Therefore, the workpiece functionality is described as its ability to meet quality standards in order to fulfill the required performance in service.

For example, the critical components of an aircraft must be manufactured under high specifications of dimensional accuracy, surface finishing, and mechanical properties. In particular, the turning of aluminum alloy pieces by removing cutting fluids increases its environmental performance. This implies a loss of surface integrity that compromises safety and therefore functionality [1,6,7,8], as dry turning is a very aggressive process that enables tool wear or more specifically, secondary adhesion. This kind of wear involves the addition of machined material to the edge and to the rake face of the tool, giving rise to the so-called built-up edge (BUE) and built-up layer (BUL), respectively [9]. 

Additionally, functional properties of manufactured elements can be changed by the action of its environment. This action can be more or less intense depending on the surface state of the manufactured element. Therefore, in the case of saline environments, corrosion depends on the surface finishing of the worked elements [10] and, consequently, on the manufacturing process. In these cases, the influence of the corrosion damage on the surface properties of the workpieces must be taken into account [11]. This is the case of different structural elements of aircrafts, especially transoceanic ones. All considered, in order to approach conditions of the actual service, it is necessary to research the influence of manufacturing process on mechanicals properties in conjunction with a corrosion environment. However, to our knowledge, there are no studies in the current literature that consider the salinity effect and its relationship with the machining process and the functional performance of the workpiece. For this reason, this paper analyses the influence of turning processes in the surface integrity of UNS A92024-T3 alloys, before and after corrosion by a saline atmosphere. More specifically, the ultimate tensile strength (UTS) is measured as a reference parameter to assess the functional performance of the material under corrosion.

## 2. Materials and Methods 

**Figure 1:**
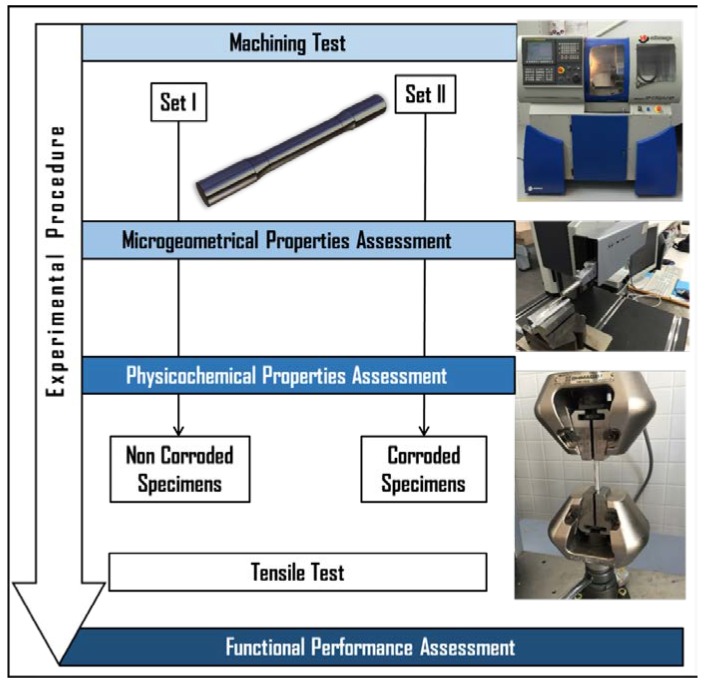
Experimental methodology scheme.

An experimental methodology was designed to achieve the proposed goal (Figure 1).

**Figure 2:**
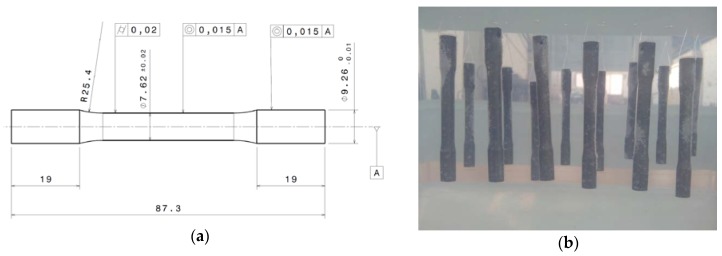
(**a**) Specimen dimensions according to standard UNE-EN ISO 6892-1:2016. (**b**) Set II samples during a corrosion test by immersion.

Al-Cu alloy UNS A92024-T3 specimens (composition in Table 1 [12]) were machined using a CNC lathe Eclipse from Alecop (Mondragón, Spain) (Figure 1). Specimens were designed according to ISO 6892-1:2016 (Figure 2a) [13]. The entire machining process was performed in absence of cooling fluids, therefore improving environmental performances.

Blocks of 32 specimens—divided in two equal sets of 16 pieces—were dry machined for this study. Workpieces for Set I were only dry turned before being tensile tested, while samples for Set II were exposed to corrosion after being dry turned and before being tensile tested. 

Machining procedure of all specimens involved a roughing process using a cutting speed (*Vc*) of 80 m/min, a feed rate (*f*) of 0.03 mm/min and a cutting depth (*d*) of 0.50 mm as cutting parameters. The finishing pass of the sample surfaces were carried out in dry conditions and using a new tool for each machined specimen with the cutting parameters shown in Table 2. 

The cutting tools used were neutral interchangeable insert (WC-Co) with commercial reference SECO, ref. DCMT 070208-F2 HX (Seco Tools AB, Fagersta, Sweden).

The surface microgeometry of the samples was evaluated through the average surface roughness parameter (*Ra*) according to the standard ISO 4288:1996 [14]. Four profiles were acquired in four equidistant generatrices for each sample using a Mahr Perthometer M1 profilometer (Mahr GmbH, Göttingen, Germany). Each specimen *Ra* was calculated as the mean value of the four *Ra* of the measured profiles.

Next, Set II was exposed to corrosion by immersion in a 10-L solution of distilled and deionized water and NaCl (3.5%) for 72 h at 296.15 K (Figure 2b) following standard ASTM NACE/ASTMG31-12a [15]. Water evaporation was controlled every day.

After each corrosive treatment, the workpieces were cleaned with distilled water in a similar way to overseas aircrafts.

Finally, in order to obtain the UTS, both sets underwent a tensile test with a Shimadzu Autograph AG-X (50 kN) tensile-compression machine (Shimadzu, Kyoto, Japan) for a precision within 1%. The crosshead velocity was *u* = 14.5 mm/min for all tests and the standard ISO 6892-1:2016 was used.

**Figure 3:**
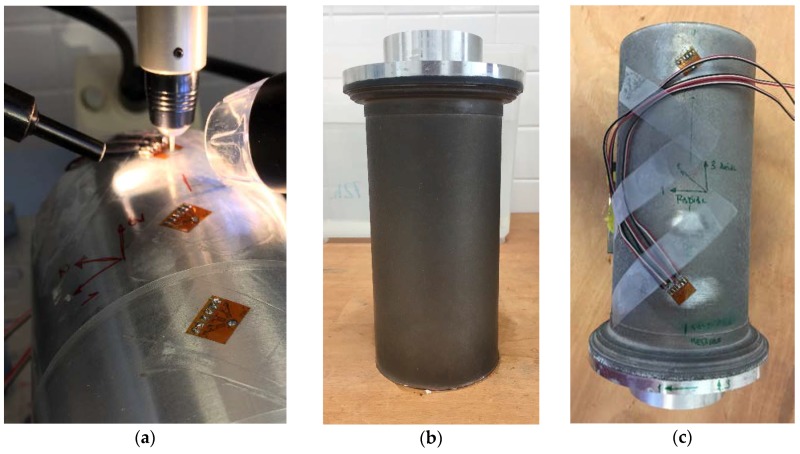
(**a**) Residual stress measurement of noncorroded specimen. (**b**) Specimen corroded after 72 h in solution of distilled and deionized water and NaCl (3.5%). (**c**) Set up of measures in corroded specimen.

On the other hand, residual stress measurements were carried out by blind hole drilling, following the ASTM E837-13a standard [16], using a RS-200 equipment from Vishay (Raleigh, North Carolina, USA) (Figure 3) [17,18]. For this purpose, CEA-13-062UM strain gages (Vishay Precision Group—Micro-Measurements, Raleigh, NC, USA) were used in this study. This method was conducted on bigger specimens with a radius of 50 mm as demonstrator, as blind hole drilling is not suitable for the 3.81 mm radii of curvature specimens. These samples followed the same turning and corrosion procedures as the original set of 32 specimens for reproducibility.

Because residual stress in machined workpieces varies with depth from the specimen surface, the integral method was used to transform strains into stresses. Measurements had a probability bound of 90%. 

## 3. Results and Discussion

**Figure 4:**
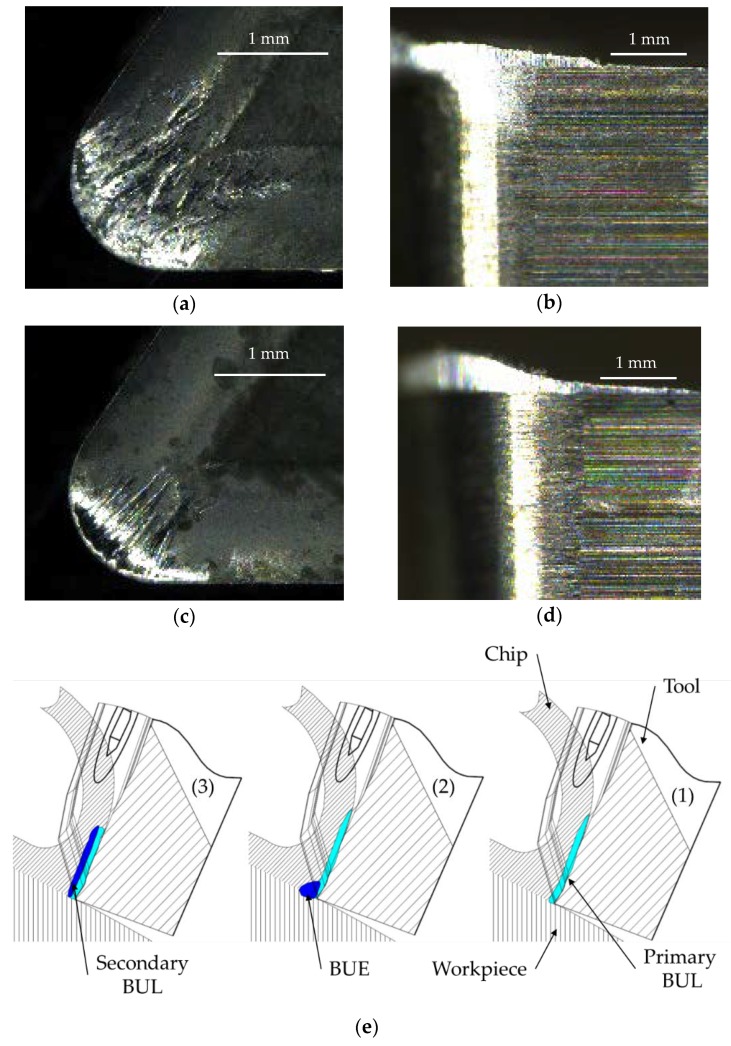
Comparison of built-up layer (BUL) and built-up edge (BUE) for different cases. (**a**,**b**) *Vc* = 100 m/min, *f* = 0.2 mm/r. (**c**,**d**) *Vc* = 40 m/min, *f* = 0.05 mm/r. (**e**) Scheme of the BUL and BUE formation.

As the time of machining is very short—ranging from 8.51 s for the shortest combination of cutting speed and feed rate to 212.74 s for the largest—no microstructural changes on the tool were expected [9,19,20,21]. However, the temperature can be high enough for softening the Al matrix and developing primary BUL. Figure 4 shows Stereoscopic Optical Microscopy (SOM) images of tools after machining under two different cutting parameters and a scheme of tool wear by secondary adhesion. 

BUL was developed onto the rake face of the tool and its size was bigger when cutting speed increased (Figure 4a,c). Primary BUL was formed in the first 5 to 10 s of machining, and it was formed by pure aluminum (Figure 4e (1)) [9]. Tool changes facilitated the mechanical adhesion of the machined alloy, giving rise to BUE (Figure 4b,d). BUE was formed by the alloy material and it grew to a critical size (Figure 4e (2)) [1,9]. When the temperature was sufficiently high, BUE softened and extruded onto the rake face, giving rise to a secondary BUL (Figure 4e (3)) [1,9]. The temperature in the cutting region was higher for increasing cutting speeds [7]. This explains the aforementioned BUL thickness. According to that, a lower BUE thickness was detected when lower cutting speed was applied. On the other hand, a higher feed involved a higher lateral chip compression, facilitating BUL through the increase of temperature caused by the relaxation process after deformation [22].

**Figure 5:**
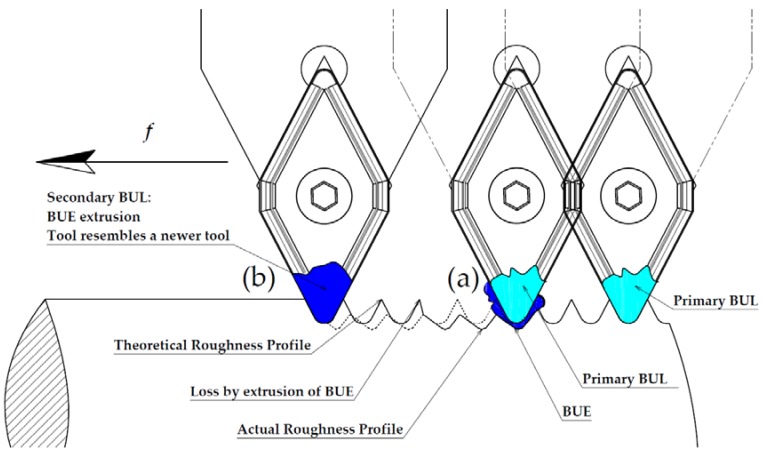
Effect of tool wear by secondary adhesion in the workpiece roughness profile for a horizontal dry turning process.

From geometric considerations, *Ra* depends directly on *f* and the edge position angle of the tool for horizontal turning processes [1,9]. The BUE development diminished this angle and, consequently, the height of the peaks in the profile reduced and smoothened, thereby decreasing *Ra* (Figure 5a). This shows that the effect of the tool wear seems to be responsible for a decrease in *Ra* in certain sets of parameters. Secondary adhesion is a dynamic process, that is to say, the morphology of the tool can change at any time (Figure 5a). In this sense, when BUE was extruded, the height of peaks increased and so did *Ra* (Figure 5b) [1,9].

**Figure 6:**
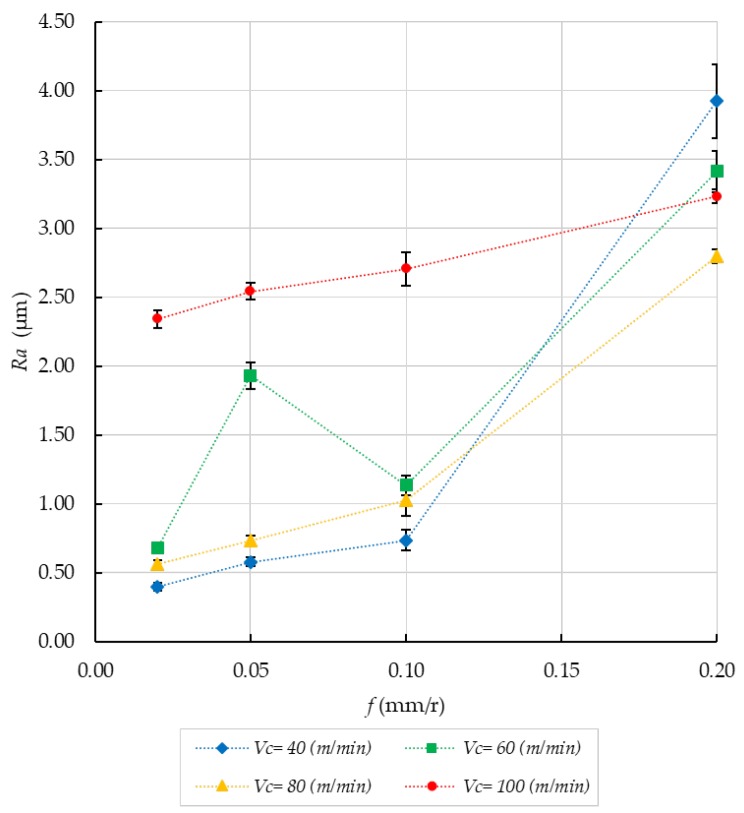
Evolution of *Ra*/*f* for all the cutting speeds studied. Error bars show the statistical standard deviation.

According to this, the extreme value of *Ra* found in (*Vc*, *f*) = (60, 0.05) can be explained (Figure 6). On the other hand, despite dry turning significantly shortening the tool life, it may have a positive effect on the microgeometrical properties of the specimen, at least in a controlled length of machining [23]. In fact, surface integrity got worse as feed increased for every tested cutting speed, as expected (Figure 6). These results are in good agreement with previous studies [19,24].

**Figure 7:**
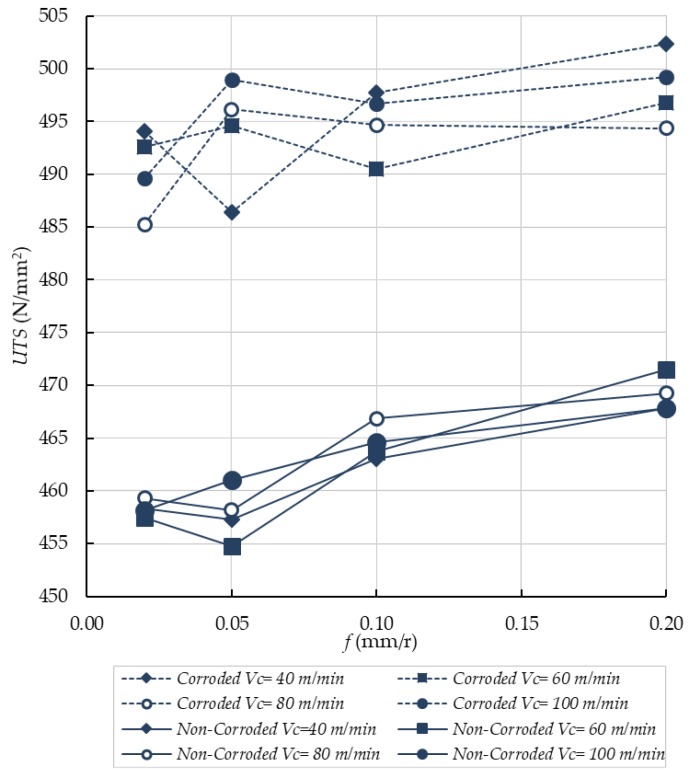
Changes in UTS/*f* for all the analyzed cutting speeds. Set I, noncorroded specimens (continuous lines). Set II, corroded specimens (dotted lines).

Figure 7 shows how the UTS increased with the feed rate for each tested specimen.

We can observe that the machining process enhanced the tensile strength for all the studied cases. In fact, the standardized value of UTS for UNS A92024-T3 lies between 440 and 450 N/mm^2^ [12,25,26]. Although the UTS variation is less than 4% of the reference value for this alloy, it is worth noting that the best results are achieved for higher feed conditions. Our findings show that higher *Ra* results in higher UTS. This can suggest that physicochemical properties of the material prevail over microgeometrical properties for surface integrity functional performance [27,28,29]. 

**Figure 8:**
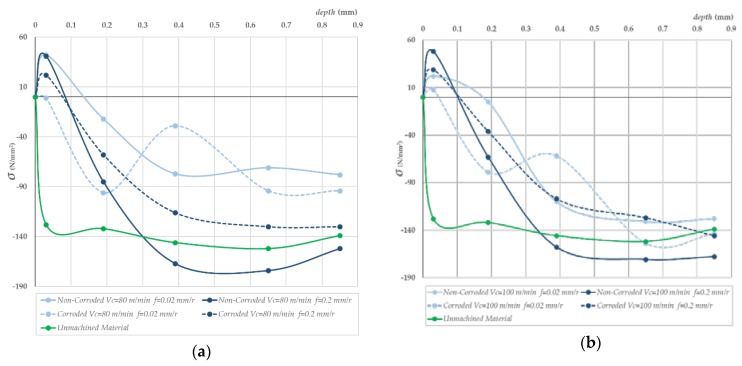
Evolution of axial residual stress/depth after machining for the cutting parameters indicated in noncorroded and corroded specimens. (**a**) *Vc* = 80 mm/min, (**b**) *Vc* = 100 mm/min. Two feed rates shown in both cases, *f* = 0.02 mm/r and *f* = 0.2 mm/r.

**Figure 9:**
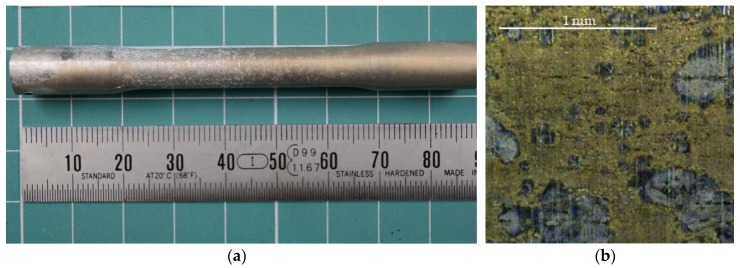
(**a**) General image of corroded specimen (*Vc* = 60 m/min, *f* = 0.2 mm/r). (**b**) Detail (×72) of the corroded surface.

As expected, higher feed rates resulted in higher compressive stresses in the surface of the specimens (Figure 8) [29,30,31,32]. However, the stress distribution was not homogeneous. The region between 0.1 to 0.9 mm was under compression, while tensile stresses were located in the first 0.1 mm of the surface where the corrosion process took place. In fact, corrosion of Al-Cu alloys in aerated NaCl solutions is complex. As a first step, the Cu of anodic intermetallics is dissolved, changing their character to a cathodic behavior. The rest of intermetallics are cathodic to the Al matrix and therefore OH^−^ is produced in the surrounding of those intermetallics. As a result, the metal matrix is dissolved by alkaline action, provoking them to fall. This process is known as Localized Alkaline Corrosion (LAC), and it does not promote the presence of cracks onto the alloy surface [11,33]. At macroscopic scale, a preferential attack cannot be seen. Moreover, different mechanisms are responsible for the deterioration [33], although the final results are homogeneously distributed onto the surface. Therefore, no pitting is developed on the specimen surfaces (Figure 9). For this reason, corrosion only affects the first layers of material, removing the tensile stress region and therefore enhancing the functional performance by a significant increase in the UTS for each workpiece, (Figure 7).

Figure 8 shows the behavior of the axial residual stress with depth in the material surface. Axial residual stresses must be taken into account because they contribute to the stress carried out by the tensile tests. Furthermore, microgeometrical defects are disposed perpendicularly to the tensile strength, so any compressive stress will tend to close surface defects, improving the functional performance [29,34]. In addition, the level of compressive residual stress increases with feed for noncorroded specimens, strengthening the compressive residual stress that the unmachined material originally supports [30,31,32]. By contrast, for decreasing feeds, compressive residual stresses are lower than that of the unmachined specimens. This is to say, functional performance is improved at higher feeds.

In summary, corrosion removed the tensile stress region at the surface of the workpieces, improving the functional performance. However, an accurate control of the corrosion process is needed because the corrosive process can have an impact in the intermetallic loss, these particles being responsible for the alloy strength [33].

## 4. Conclusions

A novel approach to study the influence of turning processes in the UTS performance after corrosion of the UNS A92024-T3 alloy has been carried out. From analysis of the results, the conclusions can be summarized as follows:

1. Machining process can improve the tensile strength of horizontal dry turned samples of aeronautical alloy UNS A92024-T3. In this limited context, functional performance is favored by machining. Physicochemical properties are responsible for improving the mechanical properties and hence the functional performance.
2. Generally speaking, the UTS increases with the feed. Thus, there is no predominant influence of the microgeometrical properties acquired after machining over the UTS. In this sense, tensile residual stress taking place on the surface after machining is not large enough to generate a decrease of UTS value.
3. The compressive residual stress after machining is responsible for the best results of UTS. Furthermore, as the feed increases, the compressive residual stress increases too, thereby improving the value of compressive residual stress of the unmachined material. Thus, the higher the compressive residual stress, the higher the UTS value.
4. The results of the test of tensile stress after corrosion show a generalized improvement of the UTS value. The corrosion process removes the first layers of material. These layers, as shown in the results, carry a tensile residual stress and are softer than the unmachined material.

## Figures and Tables

**Table 1:** Composition of aluminum-copper alloy UNS A92024 (% weight).

Cu	Mg	Mn	Si	Fe	Zn	Ti	Cr	Al
4.0	1.5	0.6	0.5	0.5	0.25	0.15	0.10	Rest

**Table 2:** Cutting parameters performed in machining test for a total of 16 experiments.

*Vc* (m/min)	*f* (mm/r)	*d* (mm)
40	0.02	0.50
60	0.05	0.50
80	0.10	0.50
100	0.20	0.50
